# Surgical treatment for hepatocellular carcinoma with portal vein tumor thrombus: a novel classification

**DOI:** 10.1186/s12957-015-0493-x

**Published:** 2015-02-28

**Authors:** Jiang-feng Xu, Xi-yu Liu, Shuai Wang, Huai-xi Wen

**Affiliations:** Department of Surgery, The Fourth Affiliated Hospital of Zhejiang, University School of Medicine, East building in Huajiachi campus, Kaixuan road 268, Hangzhou, Zhejiang 310020 China

**Keywords:** Surgical treatment, Hepatocellular carcinoma, Portal vein tumor thrombus, Novel classification

## Abstract

**Background:**

The hepatic resection for hepatocellular carcinoma (HCC) with portal vein tumor thrombus (PVTT) which is not uncommon at clinic continues to be debated. Our study introduced a novel classification of HCC with PVTT and compared the outcomes of surgical treatment between different groups.

**Methods:**

From January 2008 to December 2012, a total of 56 cases of HCC with PVTT underwent liver resection combined with thrombectomy. Clinical pathological features and surgical data of these patients were retrospectively studied. The patients were divided into two groups. Cumulative overall and disease-free survival curves of the patients were compared according to different groups.

**Results:**

Sixteen patients (28.6%) belonging to group A were compared to 40 patients (71.4%) belonging to group B. The rates of capsular formation and tumor number showed differences between the two groups (*P* = 0.047, *P* = 0.032). Group A had more liver cirrhosis than group B (*P* = 0.047). The patients with large blood loss (≥1,000) were more in group A, as well. There was no significant difference in complications between the two groups except the ascites (*P* = 0.028). The 1-year overall survival rate of group A after liver resection was 31.5%. The 1-, 3-, and 5-year overall survival rates of group B were 62.3%, 16.1%, and 5.2%, respectively. For further study, group B had significantly better overall survival than group A (*P* = 0.033). Group A had significantly higher incidence of recurrence than group B (*P* = 0.021).

**Conclusions:**

Liver resection combined with thrombectomy for HCC with PVTT can get better outcome in the HCC patients with PVTT involving only one branch (left/right) of portal vein (group B) compared to patients with PVTT involving the main portal vein trunk or both the left and right portal veins (group A).

**Electronic supplementary material:**

The online version of this article (doi:10.1186/s12957-015-0493-x) contains supplementary material, which is available to authorized users.

## Background

Hepatocellular carcinoma (HCC) is one of the most common malignancies worldwide which ranks as the second leading cause of cancer death among males [1-4]. In China, HCC ranks as the second most frequent cause of cancer death in males [5-7]. Despite the progress in surgical techniques, the prognosis of HCC especially HCC with portal vein tumor thrombus (PVTT) remains unsatisfactory. PVTT is one of the most significant factors for poor prognosis. The median survival of untreated HCC with PVTT was reported to be 2.7 months. The treatment to the HCC with PVTT is still controversial [8-11]. Although liver resection combined with thrombectomy was considered to be a valid therapeutic technique for these patients, the efficiency of surgical treatment for HCC with PVTT still remains to be evaluated [12-15].

## Methods

From January 2008 to December 2012, 56 patients with HCC complicated by PVTT underwent hepatic resection combined with thrombectomy at the Affiliated Hospital of Zhejiang University, School of Medicine, Hangzhou, China. Clinical pathological features and surgical data of these patients were retrospectively studied. Database about surgery were extracted retrospectively. The patients were divided into two groups: group A, tumor thrombi involving the main portal vein trunk or both the left and right portal veins and group B, tumor thrombi involving only one branch (left or right) of portal vein. Cumulative overall survival curves of patients were compared according to the different groups. All patients had preoperative evaluation including blood biochemistry, chest radiography, liver and renal function tests, ultrasonography, and computed tomography.

Liver resection was undertaken in the presence of good cardiopulmonary and renal function, Pugh-Child’s grades A or B, no ascites, tumor confined to one side of the liver, and no evidence of extrahepatic metastasis. Usually, we reserve the remnant liver volume more than 50% in the cirrhosis patients and 35% to 40% in noncirrhosis patients. Hepatic inflow occlusion was used routinely by Pringle’s maneuver. The liver resection was combined with the extraction of tumor thrombus through the broken ends of portal vein or by opening the trunk of portal vein. Any small PVTT in the tiny branches was suctioned. All intraoperative and postoperative complications were recorded retrospectively. Follow-up after surgery included AFP level and ultrasonography (or CT scan).

Our research was in compliance with the Helsinki Declaration. Our research have been performed with the approval of the ethics committee of the fourth Affiliated Hospital of Zhejiang University, School of Medicine.

Continuous variables are expressed as mean ± SD and compared using the independent samples *t* test. Overall survival was calculated by using the Kaplan-Meier survival method and compared using the log-rank test. All statistical analyses were performed using statistical software (SPSS 13.0 for Windows, SPSS, Chicago, IL, USA). *P* < 0.05 was considered to be statistically significant.

## Results

### Clinicopathologic features of 56 HCC patients with PVTT

**Figure 1:**
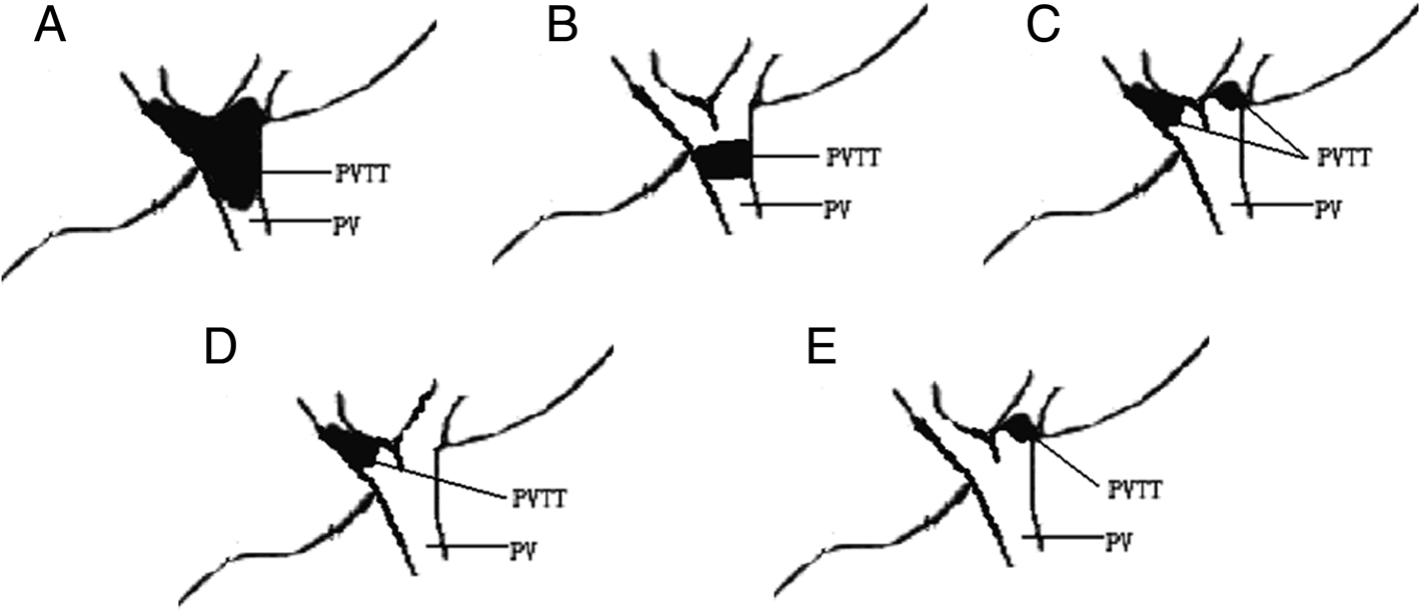
**Different classifications of PVTT. (A)**, **(B)**, and **(C)** belong to group A; **(D)** and **(E)** belong to group B. **(A)** Tumor thrombi involving the main portal vein trunk, the left portal vein and the right portal vein; **(B)** tumor thrombi involving the main portal vein trunk; **(C)** tumor thrombi involving both the left and right portal veins; **(D)** tumor thrombi involving only the right branch of portal vein; and **(E)** tumor thrombi involving only the left branch of portal vein. PV, portal vein; PVTT, portal vein tumor thrombus.

Sixteen patients (28.6%) belonging to group A were compared to 40 patients (71.4%) belonging to group B. The new classification for HCC with PVTT was shown in Figure 1. The clinical and pathologic parameters of the two groups were shown in Table 1. There were 10 patients (62.5%) that had positive hepatitis B in group A compared to 26 patients (65%) in group B. Thirteen patients (81.2%) had liver cirrhosis in group A while in group B, only 21 patients (52.5%) had liver cirrhosis. Group A had significantly more liver cirrhosis patients than in group B (*P* = 0.047). The rates of capsular formation and tumor number showed differences between two groups (*P* = 0.047, *P* = 0.032). The capsular formation in group A was 18.8% compared to 47.5% in group B. The tumor size in group A was 6.5 ± 5.0 while in group B, it was 5.3 ± 4.3.

**Table 1:** **Clinicopathologic features of 56 HCC patients with PVTT**

**Variables**	**Group A (** ***n*** **= 16)**	**Group B (** ***n*** **= 40)**	***P*** **value**
Gender			0.588
Female	3 (18.8%)	8 (20%)	
Male	13 (81.2%)	32 (80%)	
Age (years)			0.785
< 60	14 (87.5%)	36 (90%)	
≥ 60	2 (12.5%)	4 (10%)	
Hepatitis B status			0.860
Negative	6 (37.5%)	14 (35%)	
Positive	10 (62.5%)	26 (65%)	
Capsular formation			*0.047*
Presence	3 (18.8%)	19 (47.5%)	
Absence	13 (81.2%)	21 (52.5%)	
Tumor number			*0.032*
Solitary	2 (12.5%)	17 (35%)	
Multiple	14 (87.5%)	23 (65%)	
AFP level			0.797
Negative	7 (43.7%)	16 (40%)	
Positive	9 (56.3%)	24 (60%)	
Liver cirrhosis			*0.047*
Absent	3 (18.8%)	19 (47.5%)	
Present	13 (81.2%)	21 (52.5%)	
Child-Pugh classification			0.633
A	11 (68.7%)	30 (75%)	
B	5 (31.3%)	10 (25%)	
Tumor size (cm)	6.5 ± 5.0	5.3 ± 4.3	0.453

Italicized texts mean *P* < 0.05.

### Intraoperative and postoperative data of 56 HCC patients with PVTT

Intraoperative and postoperative data including major complications of 56 HCC patients with PVTT are shown in Table 2. Although there was no significant difference between the two groups in the time for hepatectomy and time for inflow occlusion (*P* = 0.595, *P* = 563), the patients with large blood loss (≥1,000) were more in group A than in group B (*P* = 0.042). The only hospital death was caused by uncontrolled bleeding. The hospital stay in group A was 15 ± 6 days compared to 14 ± 7 days in group B. There was no significant difference in complications between the two groups except the ascites (*P* = 0.028).

**Table 2:** **Intraoperative and postoperative data of 56 HCC patients with PVTT**

**Data**	**Group A (** ***n*** **= 16)**	**Group B (** ***n*** **= 40)**	***P*** **value**
Time for hepatectomy (min)	165 ± 60	156 ± 55	0.595
Time for inflow occlusion (min)	10.5 ± 9.5	11.3 ± 8.8	0.563
Blood loss			*0.042*
< 1,000	4 (37.5%)	22 (55%)	
≥ 1,000	12 (62.5%)	18 (45%)	
Blood transfusion (ml)			0.122
Without	4 (25%)	19 (47.5%)	
With	12 (75%)	21 (52.5%)	
Hospital death	1 (6.25%)	0	0.111
Hospital stay (days)	15 ± 6	14 ± 7	0.392
Major complications	4 (25%)	8 (20%)	0.680
Ascites	2	0	*0.028*
Wound infection	0	2	0.273
Pulmonary infection	0	2	0.273
Biliary fistula	0	1	0.460
Liver failure	0	1	0.460
Bleeding	2	2	0.386

Italicized texts mean *P* < 0.05.

### Overall and disease-free survival curves of the different groups of HCC patients with PVTT

**Figure 2:**
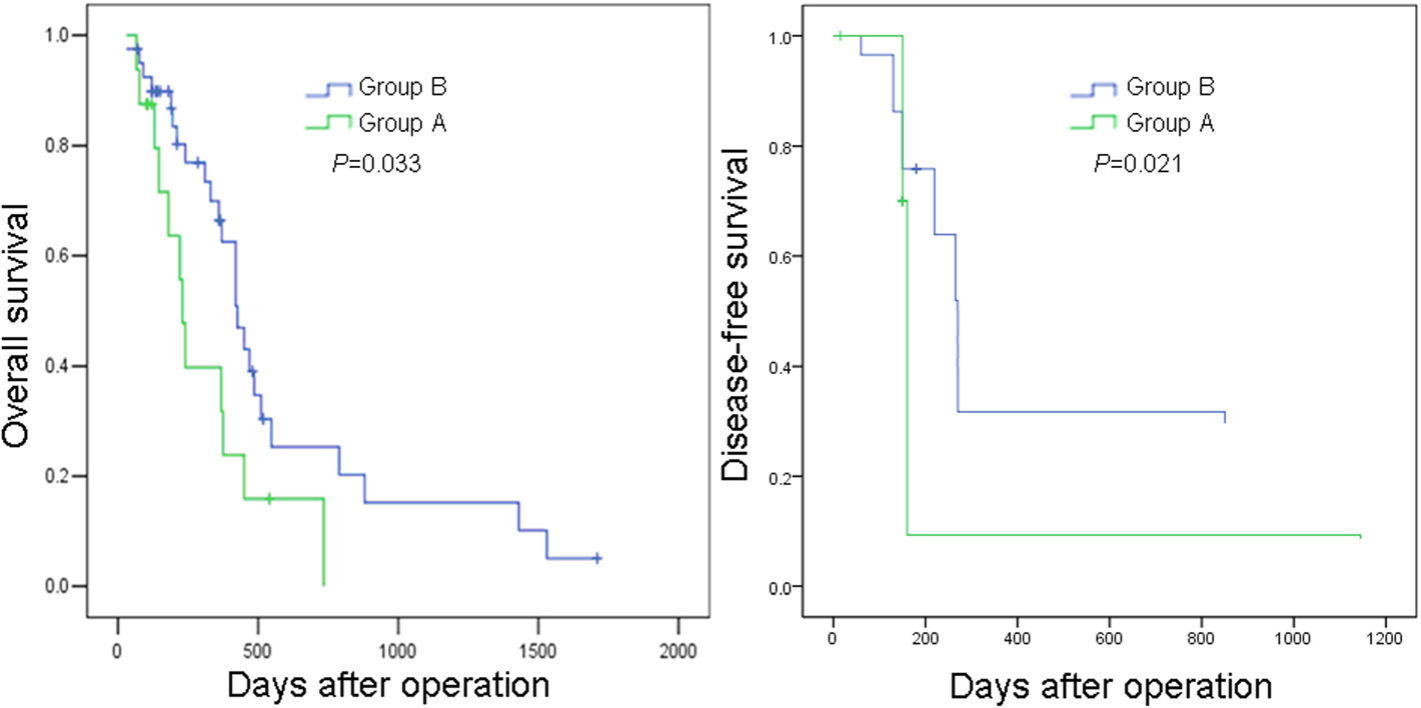
**Overall survival and disease-free survival curves of group A and group B.** Group B had significantly better overall and disease-free survival curves than group A (*P* = 0.033; *P* = 0.021).

**Figure 3:**
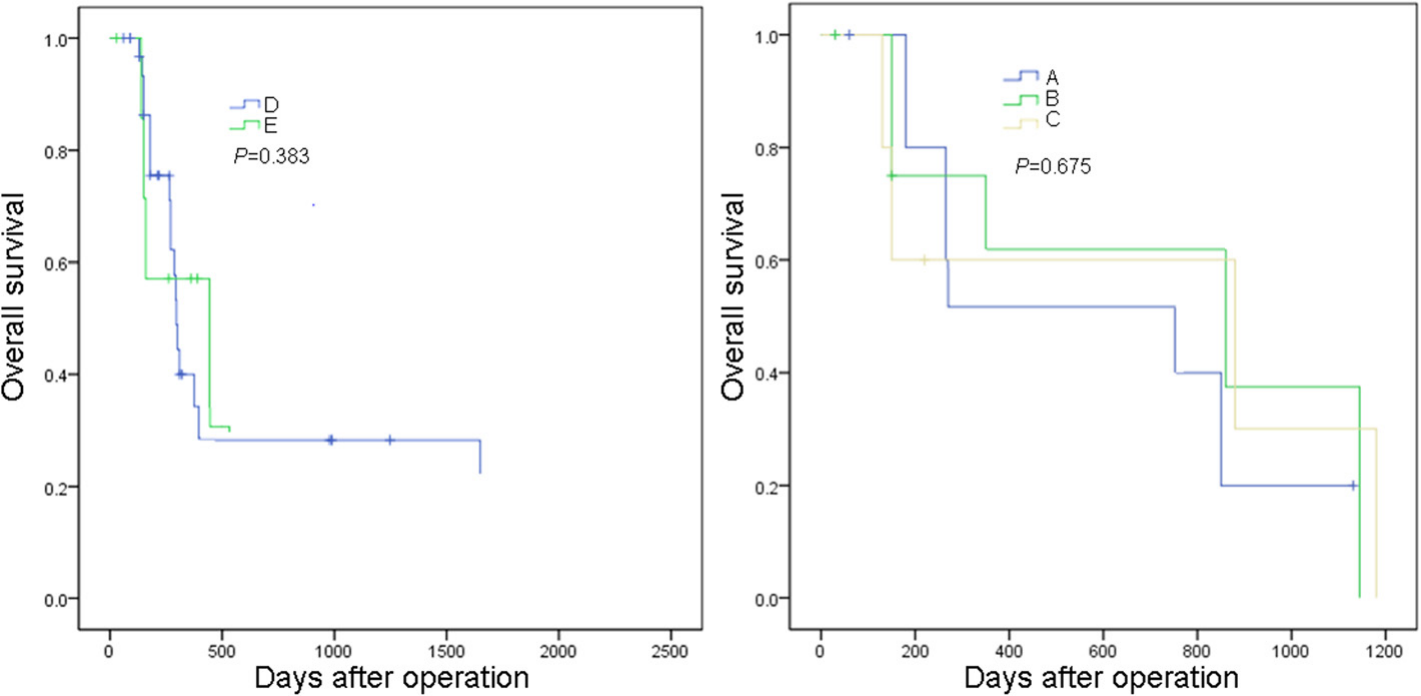
**Overall survival curves of the subgroups according to Figure** **1** **.** There was no significant difference between group A, group B, and group C (*P* = 0.675). There was no significant difference between group D and group E (*P* = 0.383).

The 1-year overall survival rate of group A after liver resection was 31.5%. The 1-, 3-, and 5-year overall survival rates of group B were 62.3%, 16.1%, and 5.2%, respectively. For further study, group B have significantly better overall survival than group A (*P* = 0.033). Group A had significantly higher incidence of recurrence than group B (*P* = 0.021) (Figure 2). We also investigated the recurrence pattern in the two groups (Additional file 1: Table S1). There was no significant difference in recurrence pattern between the two groups (*P* = 0.876). Furthermore, we investigated the overall survival curves of the subgroups according to Figure 1 (Figure 3). There was no significant difference between group A, group B, and group C (*P* = 0.675). Similarly, there was no significant difference between group D and group E (*P* = 0.383).

## Discussion

PVTT has been proven to have significant relation with HCC metastasis and recurrence after hepatectomy. HCC with PVTT was often considered to be advanced and the treatment for these patients remains controversial [16-19]. The outcome of liver resection combined with thrombectomy for 56 HCC patients with PVTT were retrospectively studied. The overall hospital death was 1.8%. This proved that the surgical treatment for HCC patients with PVTT was safe. A novel classification was introduced in this study: group A, tumor thrombi involving the main portal vein trunk or both the left and right portal veins and group B, tumor thrombi involving only one branch (left or right) of portal vein (Figure 1). Group A had more severe blood occlusion of portal vein system which may develop into portal hypertension. Interestingly, we found that group A had more liver cirrhosis than group B (*P* = 0.047). This implied that there may have been a relation between the two factors. The rates of capsular formation and tumor number showed differences between the two groups (*P* = 0.047, *P* = 0.032). As capsular formation and tumor number were reported to be the independent prognostic factors, we studied the overall survival rates of the two groups. The 1-year overall survival rates of group A after liver resection was 31.5%. The 1-, 3-, and 5-year overall survival rates of group B were 62.3%, 16.1%, and 5.2%, respectively. For further study, group B had significantly better overall survival than group A (*P* = 0.033). We also investigated the recurrence pattern in the two groups (Figure 2; Additional file 1: Table S1). Group A had significantly higher incidence of recurrence than group B (*P* = 0.021). However, there was no significant difference in recurrence pattern between the two groups (*P* = 0.876). Shi *et al*. introduced a new classification for HCC with PVTT, with their classifications; patients were classified into four types: I, II, III, and IV, each type has two subtypes [17]. In our study, patients were classified into just two groups: A and B. Both Shi’s classification and our classification were based on different prognoses of different groups. However, our classification was simpler and clearer, and Shi’s classification was more detailed and more complex.

The patients with large blood loss (≥1,000) were more in group A. This may attribute to that the extraction of tumor thrombus in group A was often by opening the trunk of portal vein. While in group B, the extraction of PVTT was often through the broken ends of portal vein. Postoperative complications of the two groups were compared. There was no significant difference in complications between the two groups except the ascites (*P* = 0.028). This may attribute to that group A had more liver cirrhosis.

## Conclusions

In conclusion, liver resection combined with thrombectomy for HCC with PVTT is safe and can get better outcome in HCC patients with PVTT involving only one branch (left/right) of portal vein compared to patients with PVTT involving the main portal vein trunk or both the left and right portal veins.

## Additional file

Additional file 1: Table S1. The recurrence pattern of the two groups.
