# Supplementary material for: Surgical treatment for hepatocellular carcinoma with portal vein tumor thrombus: a novel classification
Source: World J Surg Oncol. 2015 Feb 28;13:86. doi: 10.1186/s12957-015-0493-x (PMC4352541; doi:10.1186/s12957-015-0493-x)
Supplement: Additional file 1: Table S1. — The recurrence pattern of the two groups. [file 12957_2015_493_MOESM1_ESM.doc]

**Supplementary Table 1.**

**The recurrence pattern of two groups**

| **recurrence pattern** | **Group A** | **Group B** | ***P* value** |
| --- | --- | --- | --- |
| intrahepatic recurrence | 9 | 18 |  |
| portal thrombosis | 5 | 10 | 0.876 |
